# Control of electronic transport in graphene by electromagnetic dressing

**DOI:** 10.1038/srep20082

**Published:** 2016-02-03

**Authors:** K. Kristinsson, O. V. Kibis, S. Morina, I. A. Shelykh

**Affiliations:** 1 Division of Physics and Applied Physics, Nanyang Technological University, 637371, Singapore; 2 Department of Applied and Theoretical Physics, Novosibirsk State Technical University, Karl Marx Avenue 20, Novosibirsk 630073, Russia; 3 Science Institute, University of Iceland, Dunhagi-3, IS-107, Reykjavik, Iceland; 4 ITMO University, St. Petersburg 197101, Russia

## Abstract

We demonstrated theoretically that the renormalization of the electron energy spectrum near the Dirac point of graphene by a strong high-frequency electromagnetic field (dressing field) drastically depends on polarization of the field. Namely, linear polarization results in an anisotropic gapless energy spectrum, whereas circular polarization leads to an isotropic gapped one. As a consequence, the stationary (dc) electronic transport in graphene strongly depends on parameters of the dressing field: A circularly polarized field monotonically decreases the isotropic conductivity of graphene, whereas a linearly polarized one results in both giant anisotropy of conductivity (which can reach thousands of percents) and the oscillating behavior of the conductivity as a function of the field intensity. Since the predicted phenomena can be observed in a graphene layer irradiated by a monochromatic electromagnetic wave, the elaborated theory opens a substantially new way to control electronic properties of graphene with light.

Since the discovery of graphene^1^, it has attracted the persistent interest of the scientific community. Particularly, the influence of an electromagnetic field on the electronic properties of graphene is in the focus of attention^2^,^3^. Usually, the electron-field interaction is considered within the regime of weak light-matter coupling, where the electron energy spectrum is assumed to be unperturbed by photons. However, a lot of interesting physical effects can be expected within the regime of strong light-matter coupling, where the electron energy spectrum is strongly modified by a high-frequency electromagnetic field. Following the conventional classification, this regime is jurisdictional to quantum optics which is an established part of modern physics^4^,^5^. Therefore, the developing of interdisciplinary research at the border between graphene physics and quantum optics is on the scientific agenda.

The methodology of quantum optics lies at the basis of various exciting fields of modern physics, including quantum information^6^, polaritonics^7^, quantum teleportation^8^,^9^, quantum cryptography^10^,^11^, etc. Particularly, it allows to describe fundamental physical effects (e.g., Bose-Einstein condensation of polaritons^12^ and optical bistability^13^) and creates a basis of modern technological applications (e.g., optical logic circuits^14^, novel sources of terahertz emission^15^, and novel types of lasers^16^,^17^). Within the quantum optics approach, the system “electron + strong electromagnetic field” should be considered as a whole. Such a bound electron-field system, which was called “electron dressed by field” (dressed electron), became a commonly used model in modern physics^4^,^5^. The field-induced modification of the energy spectrum and wave functions of dressed electrons was discovered many years ago and has been studied in detail in various atomic systems^18^,^19^,^20^,^21^,^22^,^23^ and condensed matter^24^,^25^,^26^,^27^,^28^,^29^,^30^,^31^,^32^,^33^. In graphene-related research, the attention has been paid to the field-induced modification of energy spectrum of dressed electrons^34^,^35^,^36^,^37^,^38^,^39^,^40^,^41^, optical response of dressed electrons^42^, transport of dressed electrons in graphene-based p-n junctions^43^ and electronic transport through dressed edge states in graphene^44^,^45^,^46^,^47^,^48^. As to stationary (dc) transport properties of a spatially homogeneous graphene layer dressed by light, they still await detailed analysis. The present Report is aimed to fill partially this gap at the border between graphene physics and quantum optics.

## Model

**Figure 1:**
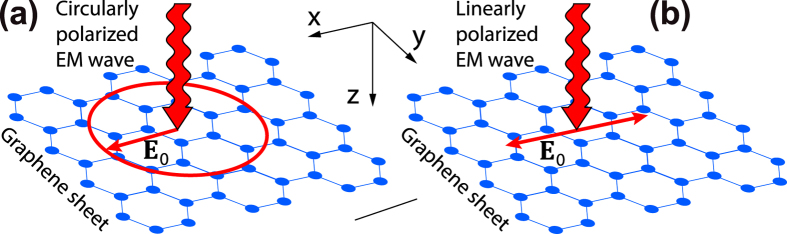
Sketch of the electron-field system under consideration. The graphene sheet dressed by (**a**) circularly polarized electromagnetic wave with the amplitude 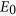 and (**b**) linearly polarized one.

For definiteness, we will restrict our consideration to the case of electron states near the Dirac point of a single graphene sheet subjected to an electromagnetic wave propagating perpendicularly to the graphene plane. Let the graphene sheet lie in the plane 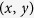 at 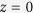, and the wave propagate along the *z* axis [see Fig. 1]. Then electronic properties of the graphene are described by the Hamiltonian^2^,^3^


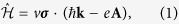


where 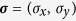 is the Pauli matrix vector, 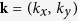 is the electron wave vector in the graphene plane, *v* is the electron velocity in graphene near the Dirac point, *e* is the electron charge, and 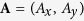 is the vector potential of the electromagnetic wave in the graphene plane. In what follows, we will be to assume that the wave frequency, *ω*, lies far from the resonant frequencies of graphene, 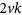. Solving the non-stationary Schrödinger equation with the Hamiltonian (1),


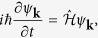


we can obtain both the energy spectrum of electrons dressed by the electromagnetic field, 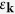, and their wave functions 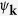 (see technical details of the solving within the Supplementary Information attached to the Report).

For the case of the circularly polarized electromagnetic field with the vector potential


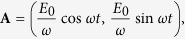


we arrive at the energy spectrum of the dressed electrons,


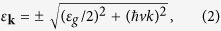


where signs “+” and “−” correspond to the conduction band and valence band of graphene, respectively,


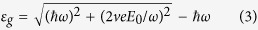


is the field-induced band gap in graphene, 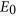 is the amplitude of electric field of the electromagnetic wave, and the field frequency *ω* is assumed to satisfy the condition of 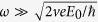. Corresponding wave functions of electrons dressed by the circularly-polarized field read as


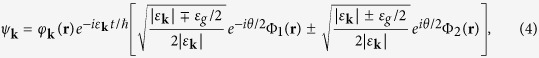


where 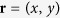 is the electron radius-vector in the graphene plane, 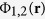 are the known basic functions of the graphene Hamiltonian (the periodical functions arisen from atomic *π*-orbitals of the two crystal sublattices of graphene)^2^, 
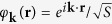
 is the plane electron wave, *S* is the graphene area, and *θ* is the azimuth angle of electron in the space of wave vector, 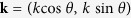.

In the case of linearly polarized electromagnetic field with the vector potential


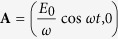


directed along the *x* axis, the energy spectrum of the dressed electrons reads as


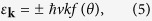


and the corresponding wave functions of dressed electrons are


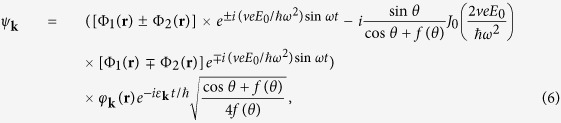


where


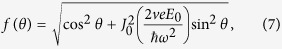



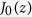
 is the Bessel function of the first kind, and the field frequency 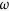 is assumed to satisfy the condition of 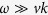.

**Figure 2:**
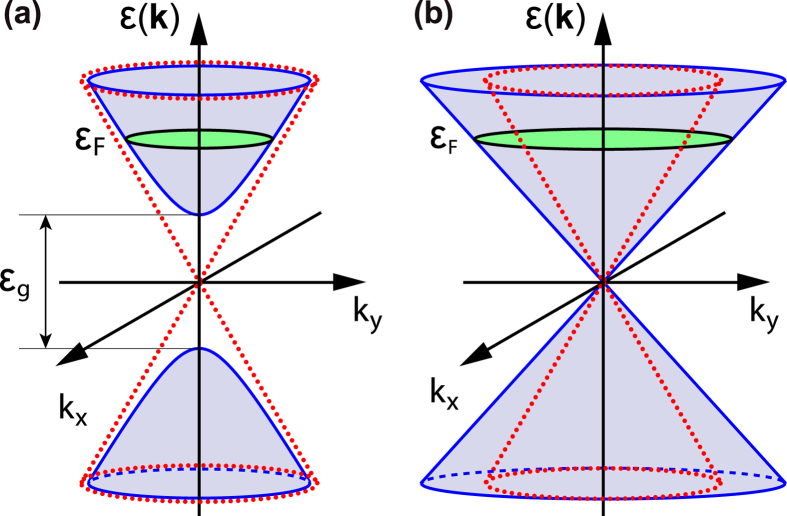
The energy spectrum of dressed electrons in graphene for the dressing field with different polarizations: (**a**) circularly polarized dressing field; (**b**) dressing field polarized along the *x* axis. The energy spectrum of electrons in absence of the dressing field is plotted by the dotted lines and 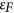 is the Fermi energy.

The energy spectra of dressed electrons, (2) and (5), are pictured schematically in Fig. 2. As to a consistent derivation of Eqs (2, 3, 4, 5, 6, 7), it can be found within the Supplementary Information attached to the Report. In order to verify the derived expressions, it should be stressed that the energy spectrum of electrons dressed by a classical circularly polarized field, which is given by Eq. (2), exactly coincides with the energy spectrum of electrons dressed by a quantized field in the limit of large photon occupation numbers^36^. This can serve as a proof of physical correctness of the presented approach elaborated for a classical dressing field.

In order to calculate transport properties of dressed electrons, we have to solve the scattering problem for nonstationary electron states (4) and (6). Following the scattering theory for dressed conduction electrons^32^, the problem comes to substituting the wave functions of dressed electrons (4) and (6) into the conventional expression for the Born scattering probability^49^. Assuming a total scattering potential in a graphene sheet, 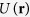, to be smooth within an elementary crystal cell of graphene, we can write its matrix elements as


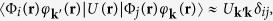


where 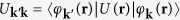, and 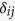 is the Kronecker delta. As a result, the Born scattering probability for dressed electronic states in graphene takes the form


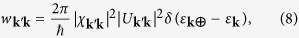


where


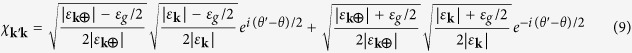


for the case of circularly polarized dressing field, and


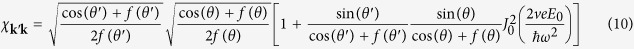


for the case of linearly polarized dressing field.

In what follows, we will assume that the wave frequency, *ω*, meets the condition


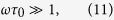


where 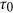 is the electron relaxation time in an unirradiated graphene, which should be considered as a phenomenological parameter taken from experiments. It is well-known that the intraband (collisional) absorption of wave energy by conduction electrons is negligibly small under condition (11) (see, e.g., Refs 32,50,51). Thus, the considered electromagnetic wave can be treated as a purely dressing field which can be neither absorbed nor emitted by conduction electrons. As a consequence, the field does not heat the electron gas and, correspondingly, the electrons are in thermodynamic equilibrium with a thermostat. Therefore, electron distribution under the condition (11) can be described by the conventional Fermi-Dirac function, where the energy of “bare” electron should be replaced with the energy of dressed electron (2),(5). Substituting both this Fermi-Dirac function and the scattering probability (8) into the conventional kinetic Boltzmann equation, we can analyze the stationary (dc) transport properties of dressed electrons in graphene. Within this approach, we take into account the two key physical factors arisen from a dressing field: (i) modification of the electron energy spectra (2) and (5) by the dressing field; (ii) renormalization of the electron scattering probability (8–10) by the dressing field.

## Results and Discussion

Let us focus our attention on the dc conductivity of the dressed electrons. Generally, the density of the conduction electrons can be tuned by applying a bias voltage which fixes the Fermi energy, 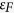, of electron gas^2^. Assuming the Fermi energy to be in the conduction band and the temperature to be zero, let us apply a stationary (dc) electric field 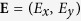 to the graphene sheet. It follows from the conventional Boltzmann equation for conduction electrons (see, e.g., Refs 2,52) that the electric current density, **J**, is given by the expression


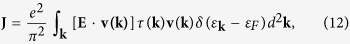


where 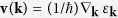 is the electron velocity, and 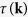 is the relaxation time. In the most general case of anisotropic electron scattering, this relaxation time is given by the equation^53^


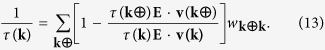


Substituting the scattering probability of dressed electron (8) into Eq. (13), we can obtain from Eqs (12,13) the conductivity of dressed graphene, 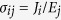.

**Figure 3:**
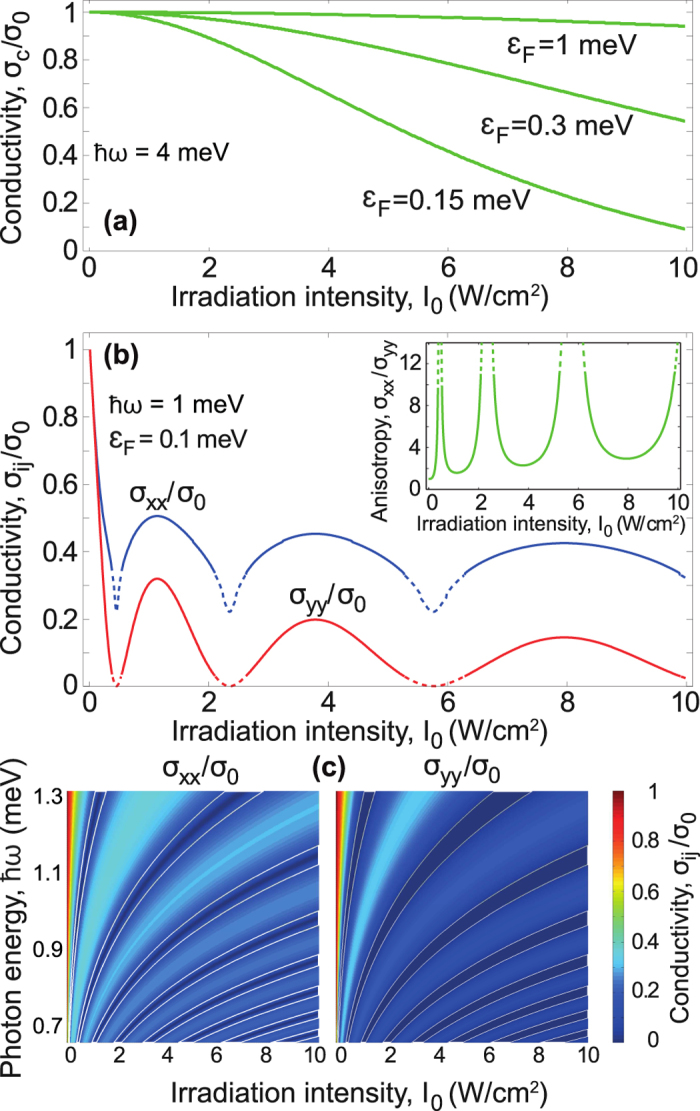
The conductivity of dressed electrons in graphene for the dressing field with the different polarizations: (**a**) circularly polarized dressing field; (**b**,**c**) dressing field polarized along the *x* axis. Physically relevant regions of the field parameters, where the developed theory is applicable, correspond to the solid lines in the plot (**b**) and wide areas between the dashed lines in the plot (**c**).

To simplify calculations, let us consider the electron scattering within the *s*-wave approximation^49^, where the matrix elements 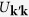 do not depend on the angle 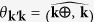. Substituting the probability (8) into Eqs (12,13), we arrive at the isotropic conductivity of a graphene dressed by a circularly polarized field, 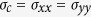, which is given by the expression


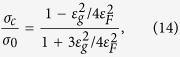


where 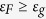. It is seen in Fig. 3a that the conductivity (14) monotonically decreases with increasing field intensity 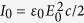. Physically, this behavior is a consequence of decreasing Fermi velocity, 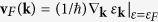, with increasing field amplitude 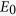 (see Fig. 2a). For the case of a dressing field linearly polarized along the *x* axis, the conductivity is plotted in Fig. 3b,c. There are the two main features of the conductivity as a function of the dressing field intensity: Firstly, the conductivity oscillates, and, secondly, the giant anisotropy of the conductivity, 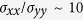 appears (see the insert in Fig. 3b). The oscillating behavior arises from the Bessel functions which take place in both the energy spectrum (5) and the scattering probability (8). As to the conductivity anisotropy, it is caused by the field-induced anisotropy of the energy spectrum (5). Namely, the linearly polarized dressing field turns the round (isotropic) Fermi line of unperturbed graphene into the strongly anisotropic ellipse line (see Fig. 2b). As a result, the Fermi velocities of dressed electrons along the 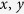 axes are strongly different and the discussed anisotropic conductivity appears. It should be stressed that the aforementioned features of electronic properties are typical exclusively for linear electron dispersion and, correspondingly, do not take place in a dressed electron gas with parabolic dispersion^33^. To avoid misunderstandings, it should be noted also that the zeros of conductivity in Fig. 3b lie within physically irrelevant areas pictured by dashed lines. Formally, these irrelevant areas correspond to the broken condition 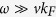, which is crucial for the correctness of the energy spectrum (5) at the Fermi energy. Thus, the zeros have no physical meaning and should be ignored.

It is seen in Fig. 3 that the behavior of conductivity is qualitatively different for the dressing field with different polarizations. Physically, the strong polarization dependence of electronic transport follows directly from the strong polarization dependence of energy spectrum of dressed electrons. Namely, the energy spectrum of electrons dressed by a circularly polarized field (2) is isotropic and has the field-induced gap (3) at the Dirac point. In contrast, the energy spectrum of electrons dressed by the linearly polarized field (5) is gapless and has the field-induced anisotropy arisen from the Bessel function in Eq. (7). These differences in the spectra (2) and (5) lead to the discussed difference of transport for electrons dressed by circularly polarized light and linearly polarized one. It should be noted that an electromagnetic field can open energy gaps within conduction and valence bands at electron wave vectors 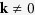 (see, e.g., Refs 38, 39, 40, 41). These gaps arise from the optical (ac) Stark effect and take place at resonant points of the Brillouin zone, where the condition of 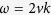 is satisfied. Certainly, the basic expressions (2–7) are not applicable near the Stark gaps. However, these gaps lie far from the Dirac point in the case of high-frequency dressing field. Therefore, they do not influence on low-energy electronic transport under consideration.

## Conclusions

We have shown that the transport properties of electrons in graphene are strongly affected by a dressing field. Namely, a circularly polarized dressing field monotonically decreases the isotropic conductivity of graphene, whereas a linearly polarized dressing field results in the oscillating behavior of the conductivity and its giant anisotropy. As a result, the dc transport properties of graphene can be effectively controlled by a strong high-frequency electromagnetic field. From the viewpoint of possible applications, the discussed effect can make graphene more tunable. Particularly, the switching times for conductivity of graphene controlled by a high-frequency field are expected to be shorter then for the case of conventional electrostatic control of conductivity by gate electrodes. This can create physical prerequisites for novel graphene-based optoelectronic devices.

## Additional Information

**How to cite this article**: Kristinsson, K. *et al*. Control of electronic transport in graphene by electromagnetic dressing. *Sci. Rep*. **6**, 20082; doi: 10.1038/srep20082 (2016).

## Supplementary Material

Supplementary Information

## References

[b1] Novoselov K. S., Geim A. K., Morozov S. V., Jiang D., Zhang Y., Dubonos S. V., Grigorieva I. V. & Firsov A. A. Electric Field Effect in Atomically Thin Carbon Films. Science 306, 666–669 (2004). 15499015 10.1126/science.1102896

[b2] Castro Neto A. H., Guinea F., Peres N. M. R., Novoselov K. S. & Geim A. K. The electronic properties of graphene. Rev. Mod. Phys. 81, 109–162 (2009).

[b3] Das Sarma S., Adam S., Hwang E. H. & Rossi E. Electronic transport in two-dimensional graphene. Rev. Mod. Phys. 83, 407–470 (2011).

[b4] Scully M. O. & Zubairy M. S. Quantum Optics (University Press, Cambridge, 2001).

[b5] Cohen-Tannoudji C., Dupont-Roc J. & Grynberg G. Atom-Photon Interactions: Basic Processes and Applications (Wiley, Weinheim, 2004).

[b6] Nielsen M. A. & Chuang I. Quantum Computation and Quantum Information (Cambridge University Press, Cambridge, 2000).

[b7] Kavokin A. V., Baumberg J. J., Malpuech G. & Laussy F. P. Microcavities (Oxford University Press, Oxford, 2007).

[b8] Bennett C. H., Brassard G., Crépeau C., Jozsa R., Peres A. & Wootters W. K. Teleporting an unknown quantum state via dual classical and Einstein-Podolsky-Rosen channels. Phys. Rev. Lett. 70, 1895–1899 (1993). 10053414 10.1103/PhysRevLett.70.1895

[b9] Bouwmeester D., Pan J.-W., Mattle K., Eibl M., Weinfurter H. & Zeilinger A. Experimental quantum teleportation. Nature 390, 575–579 (1997).

[b10] Ekert A. K. Quantum cryptography based on Bell’s theorem. Phys. Rev. Lett. 67, 661–663 (1991). 10044956 10.1103/PhysRevLett.67.661

[b11] Gisin N., Ribordy G., Tittel W. & Zbinden H. Quantum cryptography. Rev. Mod. Phys. 71, 145–195 (2002).

[b12] Kasprzak J., Richard M., Kundermann S., Baas A., Jeambrun P., Keeling J. M. J., Marchetti F. M., Szymańska M. H., André R., Staehli J. L., Savona V., Littlewood P. B., Deveaud B. & Dang L. S. Bose-Einstein condensation of exciton polaritons. Nature 443, 409–414 (2006). 17006506 10.1038/nature05131

[b13] Baas A., Karr J. Ph., Eleuch H. & Giacobino E. Optical bistability in semiconductor microcavities. Phys. Rev. A 69, 023809 (2004).

[b14] Amo A., Liew T. C. H., Adrados C., Houdré R., Giacobino E., Kavokin A. V. & Bramati A. Exciton-polariton spin switches. Nature Photon. 4, 361–366 (2010).

[b15] Kavokin K. V., Kaliteevski M. A., Abram R. A., Kavokin A. V., Sharkova S. & Shelykh I. A. Stimulated emission of terahertz radiation by exciton-polariton lasers. Appl. Phys. Lett. 97, 201111 (2010).

[b16] Christopoulos S., Baldassarri G., von Högersthal H., Grundy A. J. D., Lagoudakis P. G., Kavokin A. V., Baumberg J. J., Christmann G., Butté R., Feltin E., Carlin J.-F. & Grandjean N. Room-Temperature Polariton Lasing in Semiconductor Microcavities. Phys. Rev. Lett. 98, 126405 (2007). 17501142 10.1103/PhysRevLett.98.126405

[b17] Schneider C., Rahimi-Iman A., Kim N. Y., Fischer J., Savenko I. G., Amthor M., Lermer M., Wolf A., Worschech L., Kulakovskii V. D., Shelykh I. A., Kamp M., Reitzenstein S., Forchel A., Yamamoto Y. & Höfling. S. An electrically pumped polariton laser. Nature 497, 348–352 (2013). 23676752 10.1038/nature12036

[b18] Autler S. H. & Townes C. H. Stark Effect in Rapidly Varying Fields. Phys. Rev. 100, 703–722 (1955).

[b19] Chini M., Zhao B., Wang H., Cheng Y., Hu S. X. & Chang Z. Subcycle ac Stark Shift of Helium Excited States Probed with Isolated Attosecond Pulses. Phys. Rev. Lett. 109, 073601 (2012). 23006370 10.1103/PhysRevLett.109.073601

[b20] YuC., FuN., HuT., ZhangG. & YaoJ. Dynamic Stark effect and interference photoelectron spectra of 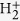 . Phys. Rev. A 88, 043408 (2013).

[b21] Kanya R., Morimoto Y. & Yamanouchi K. Observation of Laser-Assisted Electron-Atom Scattering in Femtosecond Intense Laser Fields. Phys. Rev. Lett. 105, 123202 (2010). 20867636 10.1103/PhysRevLett.105.123202

[b22] Bhatia A. K. & Sinha C. Free-free transitions of the *e*-H system inside a dense plasma irradiated by a laser field at very low incident-electron energies. Phys. Rev. A 86, 053421 (2012).

[b23] Flegel A. V., Frolov M. V., Manakov N. L., Starace A. F. & Zheltukhin A. N. Analytic description of elastic electron-atom scattering in an elliptically polarized laser field. Phys. Rev. A 87, 013404 (2013).

[b24] Goreslavskii S. P. & Elesin V. F. Electric Properties of a Semiconductor in the Field of a Strong Electromagnetic Wave. JETP Lett. 10, 316–318 (1969).

[b25] Mysyrowicz A., Hulin D., Antonetti A., Migus A., Masselink W. T. & Morkoç H. “Dressed Excitons” in a Multiple-Quantum-Well Structure: Evidence for an Optical Stark Effect with Femtosecond Response Time. Phys. Rev. Lett. 56, 2748–2751 (1986). 10033080 10.1103/PhysRevLett.56.2748

[b26] Vu Q. T., Haug H., Mücke O. D., Tritschler T., Wegener M., Khitrova G. & Gibbs H. M. Light-Induced Gaps in Semiconductor Band-to-Band Transitions. Phys. Rev. Lett. 92, 217403 (2004). 15245317 10.1103/PhysRevLett.92.217403

[b27] Dynes J. F., Frogley M. D., Beck M., Faist J. & Phillips C. C. ac Stark Splitting and Quantum Interference with Intersubband Transitions in Quantum Wells. Phys. Rev. Lett. 94, 157403 (2005). 15904187 10.1103/PhysRevLett.94.157403

[b28] Pedersen M. H. & Buttiker M. Scattering theory of photon-assisted electron transport. Phys. Rev. B 58, 12993 (1998).

[b29] Moskalets M. & Büttiker M. Floquet scattering theory of quantum pumps. Phys. Rev. B 66, 205320 (2002).

[b30] Platero G. & Aguado R. Photon-assisted transport in semiconductor nanostructures. Phys. Rep. 395, 1–157 (2004).

[b31] Kibis O. V., Slepyan G. Ya., Maksimenko S. A. & Hoffmann A. Matter Coupling to Strong Electromagnetic Fields in Two-Level Quantum Systems with Broken Inversion Symmetry. Phys. Rev. Lett. 102, 023601 (2009). 19257272 10.1103/PhysRevLett.102.023601

[b32] Kibis O. V. How to suppress the backscattering of conduction electrons? EPL 107, 57003 (2014).

[b33] Morina S., Kibis O. V., Pervishko A. A. & Shelykh I. A. Transport properties of a two-dimensional electron gas dressed by light. Phys. Rev. B 91, 155312 (2015).

[b34] López-Rodríguez F. J. & Naumis G. G. Analytic solution for electrons and holes in graphene under electromagnetic waves: Gap appearance and nonlinear effects. Phys. Rev. B 78, 201406(R) (2008).

[b35] Oka T. & Aoki H. Photovoltaic Hall effect in graphene. Phys. Rev. B 79, 081406(R) (2009).

[b36] Kibis O. V. Metal-insulator transition in graphene induced by circularly polarized photons. Phys. Rev. B 81, 165433 (2010).

[b37] Kibis O. V., Kyriienko O. & Shelykh I. A. Band gap in graphene induced by vacuum fluctuations. Phys. Rev. B 84, 195413 (2011).

[b38] Savel’ev S. E. & Alexandrov A. S. Massless Dirac fermions in a laser field as a counterpart of graphene superlattices. Phys. Rev. B 84, 035428 (2011).

[b39] Calvo H. L., Pastawski H. M., Roche S. & Foa Torres L. E. F. Tuning laser-induced band gaps in graphene. Appl. Phys. Lett. 98, 232103 (2011).

[b40] Calvo H. L., Perez-Piskunow P. M., Pastawski H. M., Roche S. & Foa Torres L. E. F. Non-perturbative effects of laser illumination on the electrical properties of graphene nanoribbons. J. Phys.: Condens. Matter 25, 144202 (2013). 23478894 10.1088/0953-8984/25/14/144202

[b41] Syzranov S. V., Rodionov Ya. I., Kugel K. I. & Nori F. Strongly anisotropic Dirac quasiparticles in irradiated graphene. Phys. Rev. B 88, 241112(R) (2013).

[b42] Zhou Y. & Wu M. W. Optical response of graphene under intense terahertz fields. Phys. Rev. B 83, 245436 (2011).

[b43] Syzranov S. V., Fistul M. V. & Efetov K. B. Effect of radiation on transport in graphene. Phys. Rev. B 78, 045407 (2008).

[b44] Gu Z., Fertig H. A., Arovas D. P. & Auerbach A. Floquet spectrum and transport through an irradiated graphene ribbon. Phys. Rev. Lett. 107, 216601 (2011). 22181902 10.1103/PhysRevLett.107.216601

[b45] Iurov A., Gumbs G., Roslyak O. & Huang D. Photon dressed electronic states in topological insulators: tunneling and conductance. J. Phys.: Condens. Matter 25, 135502 (2013). 23462425 10.1088/0953-8984/25/13/135502

[b46] Usaj G., Perez-Piskunow P. M., Foa Torres L. E. F. & Balseiro C. A. Irradiated graphene as a tunable Floquet topological insulator. Phys. Rev. B 90, 115423 (2014). 10.1103/PhysRevLett.113.266801 25615369

[b47] Foa Torres L. E. F., Perez-Piskunow P. M., Balseiro C. A. & Usaj G. Multiterminal conductance of a Floquet topological insulator. Phys. Rev. Lett. 113, 266801 (2014). 25615369 10.1103/PhysRevLett.113.266801

[b48] Dehghani H., Oka T. & Mitra A. Out-of-equilibrium electrons and the Hall conductance of a Floquet topological insulator. Phys. Rev. B 91, 155422 (2015).

[b49] Landau L. D. & Lifshitz E. M. Quantum Mechanics: Non-Relativistic Theory (Pergamon Press, Oxford, 1991).

[b50] Ashcroft N. W. & Mermin N. D. Solid State Physics (Saunders College, Philadelphia, 1976).

[b51] Harrison W. A. Solid State Theory (McGraw-Hill, New York, 1970).

[b52] Anselm A. I. Introduction to Semiconductor Theory (Prentice-Hall, New Jersey, 1981).

[b53] Sorbello R. S. On the anisotropic relaxation time. J. Phys. F 4, 503–512 (1974).

