# Supplementary material for: Control of electronic transport in graphene by electromagnetic dressing
Source: Sci Rep. 2016 Feb 3;6:20082. doi: 10.1038/srep20082 (PMC4738282; doi:10.1038/srep20082)
Supplement: Supplementary Information [file srep20082-s1.pdf]

Control of electronic transport in graphene by electromagnetic dressing

K. Kristinsson,[1] O. V. Kibis,[2,1,*] S. Morina,[1,3] and I. A. Shelykh[1,3,4]

[1]*Division of Physics and Applied Physics, Nanyang Technological University 637371, Singapore*
[2]*Department of Applied and Theoretical Physics, Novosibirsk State Technical University, Karl Marx Avenue 20, Novosibirsk 630073, Russia*
[3]*Science Institute, University of Iceland, Dunhagi-3, IS-107, Reykjavik, Iceland*
[4]*ITMO University, St. Petersburg 197101, Russia*

SUPPLEMENTARY INFORMATION:
Full and consistent derivation of the energy spectrum and wave functions of dressed electrons in graphene

Let us consider a graphene sheet which lies in the plane (x, y) at $z = 0$ and is subjected to an electromagnetic wave propagating along the z axis (dressing electromagnetic field). Then electronic properties of the graphene are described by the Hamiltonian

$$\hat{\mathcal{H}} = v\boldsymbol{\sigma} \cdot (\hbar\mathbf{k} - e\mathbf{A}), \tag{1}$$

where $\boldsymbol{\sigma} = (\sigma_x, \sigma_y)$ is the Pauli matrix vector, $\mathbf{k} = (k_x, k_y)$ is the electron wave vector in the graphene plane, v is the electron velocity in graphene near the Dirac point, e is the electron charge, and $\mathbf{A} = (A_x, A_y)$ is the vector potential of the electromagnetic wave in the graphene plane. Solving the Schrödinger equation with the Hamiltonian (1), we can find the energy spectrum of dressed electrons and their wave functions as follows.

I. Circularly polarized dressing field

For the case of circularly polarized electromagnetic wave, its vector potential $\mathbf{A} = (A_x, A_y)$ can be written as

$$\mathbf{A} = \left(\frac{E_0}{\omega}\cos\omega t, \frac{E_0}{\omega}\sin\omega t\right), \tag{2}$$

where E_0 is the electric field amplitude of the wave, and ω is the wave frequency. Then the Hamiltonian (1) is

$$\hat{\mathcal{H}} = \hat{\mathcal{H}}_0 + \hat{\mathcal{H}}_k, \tag{3}$$

where

$$\hat{\mathcal{H}}_0 = \left(-\frac{veE_0}{\omega}\right)\begin{pmatrix} 0 & e^{-i\omega t} \\ e^{i\omega t} & 0 \end{pmatrix}, \tag{4}$$

and

$$\hat{\mathcal{H}}_k = \begin{pmatrix} 0 & v(\hbar k_x - i\hbar k_y) \\ v(\hbar k_x + i\hbar k_y) & 0 \end{pmatrix}. \tag{5}$$

The nonstationary Schrödinger equation with the Hamiltonian (4),

$$i\hbar\frac{\partial\psi_0}{\partial t} = \hat{\mathcal{H}}_0\psi_0, \tag{6}$$

describes the time evolution of electron states in the Dirac point ($\mathbf{k} = 0$). The exact solutions of the equation (6) can be sought in the form

$$\psi_0 = e^{-i\alpha t/\hbar}[A\Phi_1'(\mathbf{r})e^{-i\omega t/2} + B\Phi_2'(\mathbf{r})e^{i\omega t/2}], \tag{7}$$

where $\Phi_{1,2}'(\mathbf{r})$ are the basic functions of the 2×2 matrix Hamiltonian (1), and α, A and B are the sought parameters. Substituting the wave function (7) into the Schrödinger equation (6), we arrive at the system of algebraic equations

$$\begin{aligned} A\left(\alpha + \frac{\hbar\omega}{2}\right) + B\left(\frac{veE_0}{\omega}\right) &= 0 \\ A\left(\frac{veE_0}{\omega}\right) + B\left(\alpha - \frac{\hbar\omega}{2}\right) &= 0. \end{aligned} \tag{8}$$

The condition of nontrivial solution of the system (8),

$$\begin{vmatrix} \alpha + \frac{\hbar\omega}{2} & \frac{veE_0}{\omega} \\ \frac{veE_0}{\omega} & \alpha - \frac{\hbar\omega}{2} \end{vmatrix} = 0,$$

gives the two different parameters, $\alpha = \pm\Omega/2$, where

$$\Omega = \sqrt{(\hbar\omega)^2 + \left(\frac{2veE_0}{\omega}\right)^2}. \tag{9}$$

Therefore, there are two sets of solutions of the system (27), which correspond to these two parameters and satisfy the normalization condition, $|A|^2 + |B|^2 = 1$. As a result, there are two wave functions (7),

$$\psi_0^{\pm} = e^{\pm i\Omega t/2\hbar}\left[\sqrt{\frac{\Omega \pm \hbar\omega}{2\Omega}}\Phi_1'(\mathbf{r})e^{-i\omega t/2} \pm \frac{e}{|e|}\sqrt{\frac{\Omega \mp \hbar\omega}{2\Omega}}\Phi_2'(\mathbf{r})e^{i\omega t/2}\right], \tag{10}$$

which exactly describe electron states of irradiated graphene in the Dirac point ($\mathbf{k} = 0$). Since the two wave functions (10) are the complete function system for any time t, we can seek the solution of the Schrödinger equation with the full Hamiltonian (3) as an expansion

$$\psi_{\mathbf{k}} = a^{+}(t)\psi_0^{+} + a^{-}(t)\psi_0^{-}. \tag{11}$$

Substituting the expansion (11) into the Schrödinger equation with the full Hamiltonian (3),

$$i\hbar\frac{\partial\psi_{\mathbf{k}}}{\partial t} = \hat{\mathcal{H}}\psi_{\mathbf{k}}, \tag{12}$$

we arrive at the system of two differential equations for the coefficients $a^{\pm}(t)$,

$$\begin{aligned} i\dot{a}^{+}(t) &= v\frac{e}{|e|}\left[\frac{W_0}{\Omega}(k_x\cos\omega t + k_y\sin\omega t)a^{+}(t) - (k_x - ik_y)\left(\frac{\Omega + \hbar\omega}{2\Omega}\right)e^{-i(\Omega/\hbar-\omega)t}a^{-}(t)\right. \\ &+ \left.(k_x + ik_y)\left(\frac{\Omega - \hbar\omega}{2\Omega}\right)e^{-i(\Omega/\hbar+\omega)t}a^{-}(t)\right], \\ i\dot{a}^{-}(t) &= -v\frac{e}{|e|}\left[\frac{W_0}{\Omega}(k_x\cos\omega t + k_y\sin\omega t)a^{-}(t) + (k_x + ik_y)\left(\frac{\Omega + \hbar\omega}{2\Omega}\right)e^{i(\Omega/\hbar-\omega)t}a^{+}(t)\right. \\ &- \left.(k_x - ik_y)\left(\frac{\Omega - \hbar\omega}{2\Omega}\right)e^{i(\Omega/\hbar+\omega)t}a^{+}(t)\right], \end{aligned} \tag{13}$$

where

$$W_0 = \frac{2veE_0}{\omega}$$

is the characteristic kinetic energy of rotational electron motion induced by the circularly polarized field. In what follows, we will assume that the field frequency ω is high enough to satisfy the condition

$$W_0/\hbar\omega \ll 1. \tag{14}$$

Then we have

$$\frac{W_0}{\Omega} \approx \frac{W_0}{\hbar\omega} \approx 0,\ \frac{\Omega + \hbar\omega}{2\Omega} \approx 1,\ \frac{\Omega - \hbar\omega}{2\Omega} \approx 0, \tag{15}$$

and, correspondingly, Eqs. (13) take the form

$$\begin{aligned} i\dot{a}^{+}(t) &= -v\frac{e}{|e|}(k_x - ik_y)e^{-i(\Omega-\hbar\omega)t/\hbar}a^{-}(t), \\ i\dot{a}^{-}(t) &= -v\frac{e}{|e|}(k_x + ik_y)e^{i(\Omega-\hbar\omega)t/\hbar}a^{+}(t). \end{aligned} \tag{16}$$

Let us seek solutions of Eqs. (16) as $a_c^{\pm}(t) = C^{\pm}e^{-i(\pm\Omega/2\mp\hbar\omega/2+\varepsilon_{\mathbf{k}})t/\hbar}$, where $C^{\pm}$ and $\varepsilon_{\mathbf{k}}$ are the sought parameters. Solving the system of equations (16), we arrive at

$$\varepsilon_{\mathbf{k}} = \pm\sqrt{(\varepsilon_g/2)^2 + (\hbar vk)^2}, \tag{17}$$

and

$$\varepsilon_g = \sqrt{(\hbar\omega)^2 + \left(\frac{2veE_0}{\omega}\right)^2} - \hbar\omega. \tag{18}$$

Following the conventional terminology, Eq. (17) describes the spectrum of quasienergies of electrons in graphene dressed by a circularly polarized field, where signs "+" and "−" correspond to conduction band and valence band, respectively. The two wave functions (11), which correspond to the two energy branches (17), can be written as

$$\begin{aligned}\psi_{\mathbf{k}} \;&=\; e^{-i\varepsilon_{\mathbf{k}}t/\hbar}\left[\pm\sqrt{\frac{|\varepsilon_{\mathbf{k}}|\mp\varepsilon_g/2}{2|\varepsilon_{\mathbf{k}}|}}e^{-i\theta/2}\left(\frac{e}{|e|}\sqrt{\frac{\Omega+\hbar\omega}{2\Omega}}\Phi_1'(\mathbf{r}) + \sqrt{\frac{\Omega-\hbar\omega}{2\Omega}}\Phi_2'(\mathbf{r})e^{i\omega t}\right)\right.\\ &\left.-\;\sqrt{\frac{|\varepsilon_{\mathbf{k}}|\pm\varepsilon_g/2}{2|\varepsilon_{\mathbf{k}}|}}e^{i\theta/2}\left(\sqrt{\frac{\Omega-\hbar\omega}{2\Omega}}\Phi_1'(\mathbf{r})e^{-i\omega t} \;-\; \frac{e}{|e|}\sqrt{\frac{\Omega+\hbar\omega}{2\Omega}}\Phi_2'(\mathbf{r})\right)\right],\end{aligned} \tag{19}$$

where θ is the azimuth angle of the wave vector, $\mathbf{k} = (k\cos\theta, k\sin\theta)$. Let us write the basis functions $\Phi_{1,2}'(\mathbf{r})$ in the conventional Bloch form, $\Phi_{1,2}'(\mathbf{r}) = \Phi_{1,2}(\mathbf{r})\varphi_{\mathbf{k}}(\mathbf{r})$, where $\Phi_{1,2}(\mathbf{r})$ are the periodical functions arisen from atomic π-orbitals of the two crystal sublattices of graphene, $\varphi_{\mathbf{k}}(\mathbf{r}) = e^{i\mathbf{k}\cdot\mathbf{r}}/\sqrt{S}$ is the plane electron wave, and S is the graphene area. Keeping in mind inequalities (15), we arrive from (19) to the sought wave functions of dressed electrons in the final form,

$$\psi_{\mathbf{k}} = \varphi_{\mathbf{k}}(\mathbf{r})e^{-i\varepsilon_{\mathbf{k}}t/\hbar}\left[\sqrt{\frac{|\varepsilon_{\mathbf{k}}|\mp\varepsilon_g/2}{2|\varepsilon_{\mathbf{k}}|}}e^{-i\theta/2}\,\Phi_1(\mathbf{r}) \pm \sqrt{\frac{|\varepsilon_{\mathbf{k}}|\pm\varepsilon_g/2}{2|\varepsilon_{\mathbf{k}}|}}e^{i\theta/2}\Phi_2(\mathbf{r})\right]. \tag{20}$$

II. Linearly polarized dressing field

For the case of electromagnetic wave linearly polarized along the x axis, its vector potential $\mathbf{A} = (A_x, A_y)$ can be written as

$$\mathbf{A} = \left(\frac{E_0}{\omega}\cos\omega t, 0\right). \tag{21}$$

Then the Hamiltonian (1) is

$$\hat{\mathcal{H}} = \hat{\mathcal{H}}_0 + \hat{\mathcal{H}}_k, \tag{22}$$

where

$$\hat{\mathcal{H}}_0 = \begin{pmatrix} 0 & -1 \\ -1 & 0 \end{pmatrix}\frac{veE_0}{\omega}\cos\omega t. \tag{23}$$

and

$$\hat{\mathcal{H}}_k = \begin{pmatrix} 0 & v(\hbar k_x - i\hbar k_y) \\ v(\hbar k_x + i\hbar k_y) & 0 \end{pmatrix}. \tag{24}$$

The nonstationary Schrödinger equation with the Hamiltonian (23),

$$i\hbar\frac{\partial\psi_0}{\partial t} = \hat{\mathcal{H}}_0\psi_0, \tag{25}$$

describes the time evolution of electron states in the Dirac point ($\mathbf{k} = 0$). The exact solutions of this Schrödinger equation can be sought in the form

$$\psi_0 = [A\Phi_1'(\mathbf{r}) + B\Phi_2'(\mathbf{r})]\exp\left[-i\alpha\frac{veE_0}{\hbar\omega^2}\sin\omega t\right], \tag{26}$$

where α, A and B are the sought parameters. Substituting the wave function (26) into the Schrödinger equation (25) with the Hamiltonian (23), we arrive at the system of algebraic equations

$$\begin{aligned} A\alpha + B &= 0 \\ A + B\alpha &= 0. \end{aligned} \tag{27}$$

The condition of nontrivial solution of the system (27),

$$\begin{vmatrix} \alpha & 1 \\ 1 & \alpha \end{vmatrix} = 0,$$

gives the two different parameters, $\alpha = \pm 1$. Therefore, there are two sets of solutions of the system (27), which correspond to these two parameters and satisfy the normalization condition, $|A|^2 + |B|^2 = 1$:

$$\begin{aligned} A = B &= \frac{1}{\sqrt{2}}, \\ A = -B &= \frac{1}{\sqrt{2}}. \end{aligned} \tag{28}$$

As a result, there are two wave functions (26),

$$\psi_0^\pm = \frac{1}{\sqrt{2}} \left[\Phi_1'(\mathbf{r}) \pm \Phi_2'(\mathbf{r})\right] \exp\left[\pm i \frac{veE_0}{\hbar\omega^2} \sin\omega t\right], \tag{29}$$

which exactly describe electron states of irradiated graphene in the Dirac point ($\mathbf{k} = 0$). Since the two wave functions (29) are the complete function system for any time t, we can seek the solution of the nonstationary Schrödinger equation with the full Hamiltonian (22) as an expansion

$$\psi_\mathbf{k} = a^+(t)\psi_0^+ + a^-(t)\psi_0^-. \tag{30}$$

Substituting the expansion (30) into this Schrödinger equation with the full Hamiltonian (22),

$$i\hbar \frac{\partial \psi_\mathbf{k}}{\partial t} = \hat{\mathcal{H}} \psi_\mathbf{k}, \tag{31}$$

we arrive at the system of two differential equations for the coefficients $a^\pm(t)$,

$$\begin{aligned} i\dot{a}^+(t) &= vk_x a^+(t) + ivk_y a^-(t) \exp\left[-i\frac{2veE_0}{\hbar\omega^2}\sin\omega t\right], \\ i\dot{a}^-(t) &= -ivk_y a^+(t) \exp\left[i\frac{2veE_0}{\hbar\omega^2}\sin\omega t\right] - vk_x a^-(t). \end{aligned} \tag{32}$$

It follows from the Floquet's theorem that the functions $a^\pm(t)$ can be written as

$$a^\pm(t) = e^{-i\varepsilon_\mathbf{k} t/\hbar} \widetilde{a}^\pm(t), \tag{33}$$

where $\varepsilon_\mathbf{k}$ is the electron quasienergy in the irradiated graphene (the energy spectrum of dressed electrons), and $\widetilde{a}^\pm(t)$ are the periodical functions with the period $T = 2\pi/\omega$. Let us apply the Jacobi-Anger expansion,

$$e^{iz\sin\gamma} = \sum_{n=-\infty}^{\infty} J_n(z) e^{in\gamma},$$

to the exponents in the right side of Eqs. (32) and expand the periodical functions $\widetilde{a}^\pm(t)$ into the Fourier series

$$\widetilde{a}^\pm(t) = \sum_{n=-\infty}^{\infty} c_n^\pm e^{in\omega t}.$$

Then the differential equations (32) can be transformed to the algebraic equations

$$\begin{aligned}&\left(vk_x-\frac{\varepsilon_{\mathbf{k}}}{\hbar}-n\omega\right)c_n^+ \;+\; ivk_y\sum_{m=-\infty}^{\infty}c_{n-m}^-J_m\left(-\frac{2veE_0}{\hbar\omega^2}\right)=0,\\ &\left(vk_x+\frac{\varepsilon_{\mathbf{k}}}{\hbar}+n\omega\right)c_n^- \;+\; ivk_y\sum_{m=-\infty}^{\infty}c_{n-m}^+J_m\left(\frac{2veE_0}{\hbar\omega^2}\right)=0,\end{aligned} \tag{34}$$

where $J_m(z)$ is the Bessel function of the first kind. Equations (34) can be easily solved in the case of high-frequency field satisfying the conditions

$$\hbar\omega\gg\varepsilon_{\mathbf{k}},\ \omega\gg vk. \tag{35}$$

In this simplest case, Eqs. (34) for $n\neq 0$ can be reduced to the equation

$$c_n^{\pm}\approx i\frac{vk_y}{n\omega}\sum_{m=-\infty}^{\infty}c_{n-m}^{\mp}J_m\left(\mp\frac{2veE_0}{\hbar\omega^2}\right). \tag{36}$$

Keeping in mind that $|c_n^{\pm}|\leq 1$ and $|J_n(z)|\leq 1$, Eq. (36) leads to the evident solution $c_{n\neq 0}^{\pm}\approx 0$. After substitution of this solution into Eqs. (34), the expressions (34) are reduced to the two equations

$$\begin{aligned}\left(vk_x-\frac{\varepsilon_{\mathbf{k}}}{\hbar}\right)c_0^+ + ivk_yc_0^-J_0\left(\frac{2veE_0}{\hbar\omega^2}\right) &= 0,\\ \left(vk_x+\frac{\varepsilon_{\mathbf{k}}}{\hbar}\right)c_0^- + ivk_yc_0^+J_0\left(\frac{2veE_0}{\hbar\omega^2}\right) &= 0.\end{aligned} \tag{37}$$

Solving the system of the two algebraic equations (37) accurately, we can easily obtain both the coefficients $c_0^{\pm}$ and the energy spectrum of dressed electrons,

$$\varepsilon_{\mathbf{k}}=\pm\hbar vkf(\theta), \tag{38}$$

where

$$f(\theta)=\sqrt{\cos^2\theta+J_0^2\left(\frac{2veE_0}{\hbar\omega^2}\right)\sin^2\theta}.$$

Correspondingly, the sought wave functions of dressed electrons (30) take the final form

$$\begin{aligned}\psi_{\mathbf{k}} \;=\;& \varphi_{\mathbf{k}}(\mathbf{r})e^{-i\varepsilon_{\mathbf{k}}t/\hbar}\sqrt{\frac{\cos\theta+f(\theta)}{4f(\theta)}}\Bigg([\Phi_1(\mathbf{r})\pm\Phi_2(\mathbf{r})]\,e^{\pm i(veE_0/\hbar\omega^2)\sin\omega t}\\ &-\; i\frac{\sin\theta}{\cos\theta+f(\theta)}J_0\left(\frac{2veE_0}{\hbar\omega^2}\right)[\Phi_1(\mathbf{r})\mp\Phi_2(\mathbf{r})]\,e^{\mp i(veE_0/\hbar\omega^2)\sin\omega t}\Bigg).\end{aligned} \tag{39}$$

Taking into account Eq. (38), the two conditions (35) can be reduced to the solely condition,

$$\omega\gg vk, \tag{40}$$

which describes borders of applicability of Eqs. (38) and (39).

---

* Electronic address: `Oleg.Kibis@nstu.ru`
